# Southern hemisphere deep-water stylasterid corals including a new species, *Errina labrosa* sp. n. (Cnidaria, Hydrozoa, Stylasteridae), with notes on some symbiotic scalpellids (Cirripedia, Thoracica, Scalpellidae)

**DOI:** 10.3897/zookeys.472.8547

**Published:** 2015-01-19

**Authors:** Daniela Pica, Stephen D. Cairns, Stefania Puce, William A. Newman

**Affiliations:** 1 Department of Life and Environmental Sciences, Polytechnic University of Marche, Via Brecce Bianche, 60131 Ancona, Italy; 2 Department of Invertebrate Zoology, Smithsonian Institution, Washington, D.C., 20560, U.S.A.; 3 Scripps Institution of Oceanography, La Jolla, California 92093-0202, U.S.A.

**Keywords:** Deep-water symbiosis, Scalpellidae, Stylasteridae, new species

## Abstract

A number of stylasterid corals are known to act as host species and create refuges for a variety of mobile and sessile organisms, which enhances their habitat complexity. These include annelids, anthozoans, cirripeds, copepods, cyanobacteria, echinoderms, gastropods, hydroids and sponges. Here we report the first evidence of a diverse association between stylasterids and scalpellid pedunculate barnacles and describe a new stylasterid species, *Errina labrosa*, from the Tristan da Cunha Archipelago. Overall, five stylasterid species are found to host eight scalpellid barnacles from several biogeographic regions in the southern hemisphere (Southern Ocean, temperate South America and the southern Indo-Pacific realms). There is an apparent lack of specificity in this kind of association and different grades of reaction to the symbiosis have been observed in the coral. These records suggest that the association between pedunculate barnacles and hard stylasterid corals has a wide distribution among different biogeographic realms and that it is relatively rare and confined largely to deep water.

## Introduction

Many stylasterid corals, like their shallow-water largely scleractinian counterparts (see e.g. Patton 1994, Stella et al. 2011, Hoeksema et al. 2012), are considered habitat-forming species because they contribute to the structuring of deep and shallow water coral banks (Roberts et al. 2006, Häussermann and Försterra 2007). In this context the tridimensional structure of their calcareous skeleton should enhance the complexity of the habitat, by creating refuges for a variety of mobile and sessile organisms (Braga-Henriques et al. 2010): basibionts for many other invertebrate such as annelids, anthozoans, cirripeds, copepods, cyanobacteria, echinoderms, gastropods, hydroids and sponges (Zibrowius 1981, Braga-Henriques et al. 2010, Goud and Hoeksema 2001, Pica et al. 2012, Puce et al. 2009). Species of the gastropod *Pedicularia*, considered obligate symbionts of Stylasteridae, usually assume the colour of the host colony and modify the branch coral surface where they reside (Zibrowius 1981, Goud and Hoeksema 2001, Cairns and Zibrowius 2013). Other organisms induce changes in the coral morphology and growth, like copepods that induce the formation of a gall on the coral branches (Zibrowius 1981, Buhl-Mortensen and Mortensen 2004a) and balanomorph or acorn barnacles (Zibrowius 1981) that usually are completely covered by the coral coenosteum. The presence of polychaetes on stylasterid colonies seems to occur in about 30% of the stylasterid species and frequently induces pronounced changes in the growth form and branching pattern in many species. For example, in *Inferiolabiata labiata* (Moseley, 1879) the polynoid *Polyeunoa laevis* McIntosh, 1885 induces modifications in the growing branches prior to the production of a reticulate tube in which the worm travels (Moseley 1879, Cairns 1983a). Those epibionts probably receive protection from predators, and also access to food is increased due to the tridimensional shape of the colonies (Braga-Henriques et al. 2010).

Such associations between cnidarians and other invertebrates are fairly common. Crustaceans, in particular cirripeds, are most prevalent in shallow water, the latter largely with corals having calcareous skeletons. The most notable include the burrowing barnacles or acrothoracicans (Kolbasov 2009) and thoracican coral barnacles generally belonging to the sessile balanomorph family Pyrgomatidae (Ross and Newman 1973). Such obligate, often host specific forms attain a remarkable diversity on scleractinian corals (Newman et al. 1976, Malay and Michonneau 2014), especially regarding shell modifications in species that have become nutritionally parasitic (Ross and Newman 1995).

The general situation in deep water is quite different as it is pedunculate scalpellomorphs rather than sessile balanomorphs that predominate (Newman and Ross 1971, cf. lepadomorph/balanomorph ratio). In their review of deep-water coral symbiosis, Buhl-Mortensen and Mortensen (2004b) reported on 74 host species (33 gorgonians, 29 scleractinians, seven alcyonaceans, and five antipatharians, but no stylasterids) with obligate as well as facultative associates. They conclude that Cirripedia (including their close relatives, the Ascothoracida), are the most common crustacean taxa associates of deep-water corals. While the ascothoracids range from somewhat vagile ectoparasites to highly modified gall-forming endoparasites, the scalpellomorph pedunculates have made no obvious morphological adaptations to their hosts. However, it has been shown that calices of deep-water scleractinians such as *Lophelia pertusa* (Linnaeus, 1758), can grow up around a substantial portion of the peduncle of the scalpellomorphs (Newman et al. 2002), and herein we demonstrate for the first time that the coenosteum of stylasterid corals can do likewise. Furthermore, this paper reports the first evidence of a diverse association between stylasterids and scalpellid pedunculate barnacles from several southern biogeographic regions and describes a new stylasterid species from the Tristan da Cunha Archipelago involved in this symbiosis.

## Methods

The Stylasteridae collections of several European Museums have been studied: MNA – Museo Nazionale dell’Antartide “Felice Ippolito”, Italy; Museo di Storia Naturale of Genova, Italy; BNHM – Natural History Museum of London, United Kingdom; MNHN – Muséum National d’Histoire Naturelle of Paris, France; RMNH and ZMA in Naturalis Biodiversity Center of Leiden, Nederland. A number of specimens with pedunculate barnacles on them were examined for further analyses. The coral specimens (dry or preserved in ethanol) were, largely from the South Atlantic Ocean and the Antarctic and Sub-Antarctic region, whereas *Stephanohelia* corals were from off New Caledonia (South Pacific). The morphology of the specimens and details of the associations were first examined using a stereomicroscope. Selected portions were prepared for the scanning electron microscope (SEM) and photographic analyses. Longitudinal sections of coral branches were cut with an electric grinder in order to study the internal structures. Small portions of the coral were treated with sodium hypochlorite for 10 minutes, rinsed with distilled water, and dried, and coated with gold–palladium in a Balzer Union evaporator and examined with a Philips XL20 SEM.

## Results

Among a total of about 600 stylasterid colonies observed, only 11 (<2%) belonging to five species revealed the presence of scalpellid barnacles (Table 1). The range of morphological responses of the corals to a notable diversity of barnacles was initially not fully appreciated owing to the scarcity of the material. The identification of the barnacles has been limited to illustrated specimens and while at least two subfamilies of the species-rich Scalpellidae are represented, it was not possible to identify all to species or even genus level. Nonetheless, this paper sheds considerable light on this kind of interspecific relationship and sets the stage for future taxonomic work on the barnacles involved.

**Table 1.** Five stylasterid species and the eight scalpellids associated with them.

Stylasterid corals	Scalpellomorph barnacles
*Stephanohelia* sp. New Caledonia, 550 m	scalpellid sp. 3
*Inferiolabiata spinosa* Cairns, 1991 Tristan da Cunha, 80–140m	*Arcoscalpellum* sp. 2
*Errina antarctica* (Gray, 1872) Off Falkland Islands 79–370 m	scalpellid sp.1, scalpellid sp. 2 and Ornatoscalpellum cf. gibberum
*Errina fissurata* (Gray, 1872) off Daniell Peninsula, Antarctica, 438–610 m	*Trianguloscalpellum* sp. and Ornatoscalpellum cf. vanhoeffeni
*Errina labrosa* sp. n. Tristan da Chuna, 80–140 m	*Arcoscalpellum* sp. 1

### Systematic part Phylum Cnidaria Class Hydrozoa Owen, 1843 Subclass Hydroidolina Collins & Marques, 2004 Order Anthoathecata Cornelius, 1992 Suborder Filifera Kühn, 1913 Family Stylasteridae Gray, 1847

#### Genus Stephanohelia

Taxon classificationAnimaliaAnthoathecataStylasteridae

Cairns, 1991

##### Diagnosis.

Colonies with irregular shape, all with commensal polychaetes. Branches polychotomous with gastropores exclusively in the branch axils. Coenosteal texture linear-imbricate. Gastrostyle massive. Dactylopore spines small and without dactylostyles. Male ampullae superficial.

##### Discussion.

The genus *Stephanohelia* is monospecific (Cairns 1991). The genus is easily diagnosed by its characteristic polychotomous branching and the gastropores exclusively at branch axils.

##### Type species.

*Stephanohelia praecipua* Cairns, 1991.

##### Depth range.

318–793 m.

##### Distribution.

New Zealand and New Caledonia.

#### Stephanohelia sp.

Taxon classificationAnimaliaAnthoathecataStylasteridae

Figure 1

##### Material studied.

Three colonies of sample MNHN IK 2010-152: expedition MUSORSTOM 4 N/O Vauban, Sta. CP194, 18°53’ S, 163°22’ E, New Caledonia, 550 m depth 19 September 1985 (in ethanol).

##### Description.

**Figure 1.**
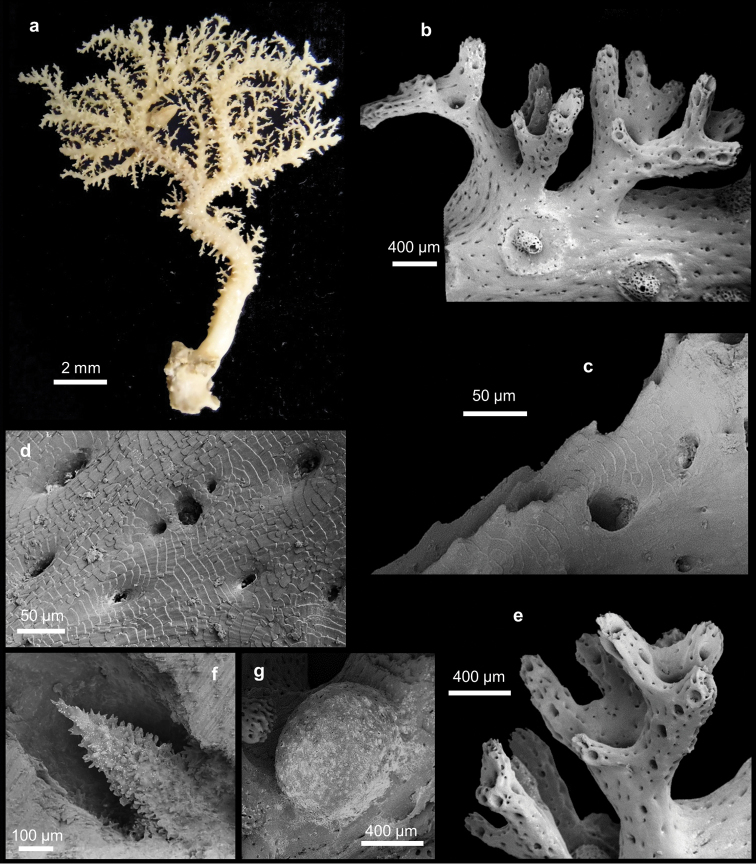
*Stephanohelia* sp. **a** Colony. SEM micrographs of **b** branch with polychotomous tiny branches and male ampullae **c** small abcauline spines **d** texture **e** polychotomous branches with aligned dactylopores **f** gastrostyle **g** female ampulla.

Coral colonies arborescent, up to 8 cm long and 7 cm wide, with the basal branches up to 1.5 cm in diameter (Figure 1a). They are characterised by few main branches that are uniplanar around which several polychotomously tiny branches originate, formed by two to five branchlets (Figure 1b). The tiny branches are characterised by small abcauline spines (Figures 1b, c), up to 20 µm tall, mainly on the lateral edges. The branches are oval in cross-section. The tiny branches on both faces of the main branches are often anastomose, forming a gallery, which is caused by a commensal polychaete (Figure 1a). The colonies are attached to the substrate by an incrusting base. The colour of the coenosteum is white (Figure 1a). The coenosteal texture is linear-imbricate, composed of platelets irregular in shape and showing an alternating polarity (Figure 1d). The coenosteum is pierced by numerous coenosteal pores, 15-22-30 µm in diameter (Figure 1d). The strips are not well defined. The coenosteum is white.

Gastropores are circular, 100-175-230 µm in diameter, occurring exclusively at branching axils (Figure 1e). The gastropore tubes are cylindrical to conical in apical branches where they are less deep. The gastrostyle tips are visible on the coenosteum surface. The gastrostyles are robust and tree-like in shape without ridges and ornamented with multi-tipped spines (Figure 1f). The gastrostyles measure 96-170-205 µm in length and 50-80-100 µm in diameter (L:D=1.8–2.6). A ring palisade is not present. The dactylopores are flush with coenosteum and aligned on branch edges (Figure 1e). They are circular in shape and 40-60-95 µm in diameter. Dactylostyles are absent.

The female ampullae are round, superficial, 600-820-900 µm in diameter, and have a smooth surface (Figure 1g). They are distributed uniformly around the branches. Male ampullae are 400-510-600 µm in diameter and characterised by a round depression with a small central dome (Figure 1b). The domes vary in shape and have a highly perforate surface, usually with an apical pore (Figure 1b).

##### Remarks.

The characteristic shape of the colonies with polychotomous branching, the presence of the gastropores exclusively at branch axils, the large gastrostyles and the absence of the ring palisade and the dactylostyles are characteristic for *Stephanohelia*. This species differs from the type species, *Stephanohelia praecipua*, mainly in the gastrostyle shape. In fact, *Stephanohelia praecipua* has a gastrostyle characterised by a main basal shaft with a very expanded midsection and a slender tip. The scarcity of the analysed material is insufficient to enable the description of a new species.

#### Genus Inferiolabiata

Taxon classificationAnimaliaAnthoathecataStylasteridae

Broch, 1951

##### Diagnosis.

Colonies commonly associated with a commensal polychaete. Gastropores and dactylopores randomly distributed. Coenosteal texture linear- or reticulate-imbricate. Gastrostyles are present but a ring palisade is usually absent. Tabulae often present. Dactylopore spines with a primarily abcauline dactylotome. Dactylostyles present. Ampullae superficial.

##### Discussion.

The genus *Inferiolabiata* includes four species (Cairns and Zibrowius 2013). The present material represents part of the first record of identified stylasterid corals from the Tristan da Cunha Archipelago.

##### Type species.

*Errina labiata* Moseley, 1879.

##### Depth range.

80–2100 m.

##### Distribution.

Tristan da Cunha Archipelago, South Africa, Antarctica and Sub-Antarctic area, New Zealand.

#### Inferiolabiata spinosa

Taxon classificationAnimaliaAnthoathecataStylasteridae

Cairns, 1991

Inferiolabiata spinosa Cairns 1991: 42; Cairns and Zibrowius 2013: 14.

##### Material studied.

BNHM 1977.8.10.2: two broken colonies and seven fragments, *Discovery* Expedition Sta. 6, Tristan da Cunha, 3 miles N 30° E of Settlement, 80–140 m depth, 1 February 1926 (in ethanol).

##### Remarks.

The genus *Inferiolabiata* consists of only four species: *Inferiolabiata labiata* (Moseley, 1879), *Inferiolabiata lowei* (Cairns, 1983a), *Inferiolabiata spinosa* Cairns, 1991 and *Inferiolabiata africana* Cairns & Zibrowius, 2013. Our specimens match *Inferiolabiata spinosa* described from New Zealand and South Africa (Cairns 1991, Cairns and Zibrowius 2013). They only differ in having longer and thinner gastrostyles (L:D up to 15), in lacking a well-defined ring palisade, and the presence of two unlinear series of dactylostyles instead of three.

This is the first record of *Inferiolabiata spinosa* from the Atlantic and together with *Errina labrosa* sp. n. (see below), it is part of the only known stylasterid fauna reported from Tristan da Cunha Archipelago.

#### Genus Errina

Taxon classificationAnimaliaAnthoathecataStylasteridae

Gray, 1835

##### Diagnosis.

Gastropores and dactylopores randomly distributed. Coenosteal texture reticulate-granular and linear-imbricate. Lower gastropore lip present in some specimens. Gastrostyles present but ring palisade usually absent. Dactylopore spines represented by up to two types and varying in shape and dimension. Dactylopore spines with a primarily adcauline dactylotome. Dactylostyles rarely present. Ampullae superficial and deep.

##### Discussion.

The genus *Errina* includes 25 Recent species and one extinct species (Cairns 2014). The presence of dactylostyles in this genus is reported here for the first time.

##### Type species.

*Millepora aspera* Linnaeus, 1767.

##### Depth range.

6–1772 m.

##### Distribution.

North Atlantic, Mediterranean Sea, Galápagos, South Africa, Antarctica and Sub-Antarctic area, New Zealand, Japan and Tristan da Cunha Archipelago.

#### Errina antarctica

Taxon classificationAnimaliaAnthoathecataStylasteridae

(Gray, 1872)

Errina antarctica See Cairns (1983a: 83) for synonymy.

##### Material studied.

BNHM 1977.8.10.20: two colonies, *Discovery* Expedition, Sta. WS 248, Falkland Islands 52°40'00"S 58°30'00"W, 210-242 m depth, 20 July 1928, (preserved in ethanol); BNHM 1977.8.10.17: five broken colonies, *Discovery* Expedition, Sta. WS 841, 54°11’S 60°23"W, 200-370 m depth, 6 February 1932 (preserved in ethanol); BNHM 1977.8.10.34: 3 broken colonies and fragments, *Discovery* Expedition, Sta. WS 85, Falkland Islands, 52°09'00"S 54°14'00"W, 79 m depth, 25 March 1927 (dry).

##### Remarks.

Within the genus *Errina* our specimens match *Errina antarctica* as described by Cairns (1983a) in all aspects of the colony morphology (see remarks of *Errina fissurata*).

#### Errina fissurata

Taxon classificationAnimaliaAnthoathecataStylasteridae

(Gray, 1872)

Figure 2

Madrepora fissurata Stokes 1847: 336
Errina fissurata Gray 1872: 745; Moseley 1879: 479; 1881: 84; Boschma 1957: 53; 1964: 284; Boschma and Lowe 1969: 15; Cairns 1983a: 89.
Labiopora fissurata Hickson 1912: 878.
Errina (Eu-Errina) fissurata Broch 1942: 38.
Errina (Eu-Errina) antarctica Broch 1951: 35 (part of material from sta. 1948).
Errina (Errina) fissurata Boschma 1963: 337; Cairns 1983a: 89.
Errina antarctica Boschma 1966: 109 (part of material from sta. 30).

##### Material studied.

MNA 3070, MNA 3071: two colonies, Cruise Carbonant 2002, Sta. 24, 72°30'456"S, 174°05'552"E, 438 m depth, 13 January 2002 (in ethanol); MNA 3079, MNA 3080, MNA 3081, MNA 3082, MNA 3086: a total of five colonies, Cruise Tangaroa 2004, Sta. 77, 72°07'47"S, 172°42'36"E, 499 m depth, 14 February 2004 (dry); BNHM 1977.8.10.26: one colony, *Discovery* Expedition, Sta. 1948, 60°49'24"S, 52°40'00"W, 490–610 m depth, 4 January 1937 (in ethanol).

##### Description.

**Figure 2.**
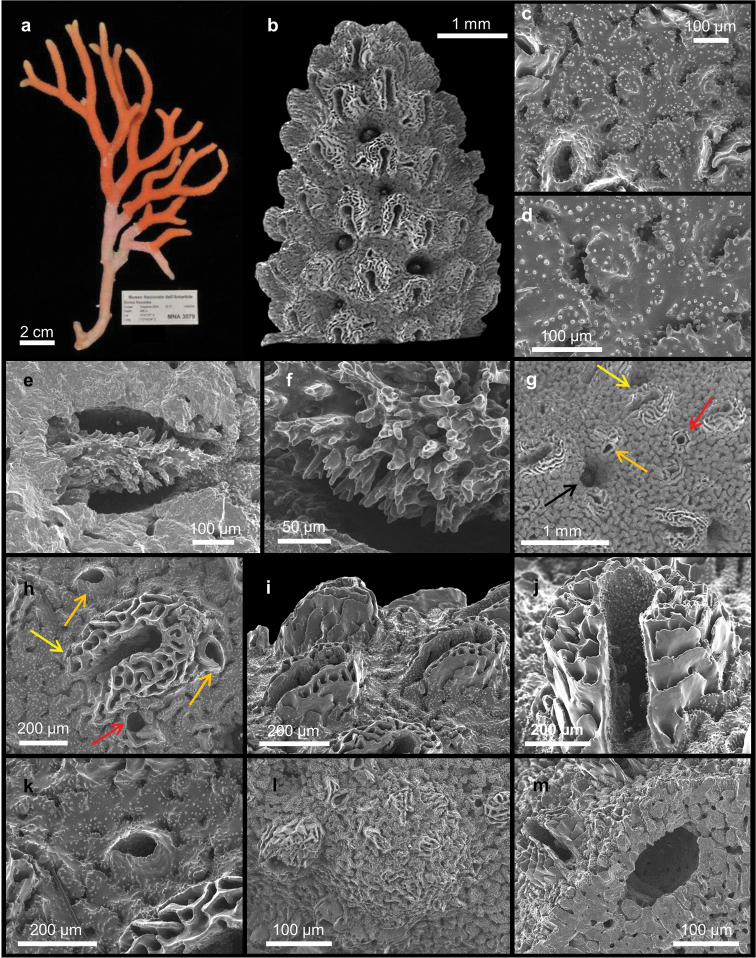
*Errina fissurata* Gray, 1872. **a** Colony. SEM micrographs of **b** apical branch **c–d** reticulate-granular coenosteal texture **e** gastrostyle **f** bifurcating spines of gastrostyle **g** different type of pores: gastropore (black arrow), large dactylopore spine (yellow arrow), small dactylopore spine (orange arrow) and large round pore (red arrow) **h** large dactylopore spine (yellow arrow), small dactylopore spines (orange arrows) and large round pore (red arrow) **i** large adcauline dactylopore spines **j** dactylostyles **k** small dactylopore spine **l** female ampulla **m** male ampulla.

Specimens consist of up to 16.4 cm long broken branches of uniplanar colonies, all lacking the base (Figure 2a). The branching pattern is irregular and dichotomous, most branches oriented nearly parallel (Figure 2a). They are oval in cross-section and up to 6 mm in diameter. The blunt tips are 2–3 mm in diameter (Figure 2b).

The colour of the coenosteum is pale orange (Figure 2a) with the core of the branches white. The texture is reticulate-granular with small and rounded granules 1.4–10 µm in diameter (Figures 2c, d). On the surface there are non-linear slits, up to 140 µm wide and provided with teeth projecting inward (Figure 2d). They connect the coenosteal pores, 20–30 µm in diameter (Figure 2d).

The gastropores and dactylopores are scattered over the coenosteum (Figure 2b). They are decreasing in abundance and density from the tip of each branch towards the base, being mainly present on the lateral branch edges and almost absent on the faces while they are generally lacking in the basal region. The round gastropores, 100–300 µm in diameter, are not lipped and the gastrostyle tips are visible from the coenosteum surface (Figure 2b). The gastrostyles are robust and lanceolate without ridges and have multi-tipped bifurcating spines (Figures 2e, f). They are up to 470 µm long and up to 220 µm in diameter (L:D=1.7–2.9). Ring palisades and tabulae are not recorded (Figure 2e).

The coenosteum surface contains two kinds of dactylopores, with either large or small spines, which protrude perpendicularly from it (Figure 2g). The large spines are U-shaped in cross-section and have an adcauline opening; they are mainly present in the distal portion of the branches (Figures 2h–j). They are 250–400 µm long with a diameter of 250–500 µm and are characterised by a thick porous wall (Figure 2h). Laterally they are composed of smooth overlapping platelets, while the internal wall is characterised by the typical reticulate texture (Figures 2i, j). The dactylotome is 60–110 µm wide (Figure 2j). From the apex to the base, the large dactylopore spines tend to become flush with the coenosteum and to disappear from the colony faces, remaining only on the lateral branch edges. The small spines are scattered between the large ones (Figures 2g, h, k), and, toward the base of the colony they become increasingly difficult to distinguish from the large dactylopores. Their external wall is smooth or reticulate and measures 60–140 × 60–85 µm in diameter and up to 70 µm in length (Figures 2h, k). The dactylotome is randomly oriented (Figure 2h). Dactylostyles are present only in the large spines (Figure 2j). They are composed by rudimentary elements up to 35 µm long and are arranged in two linear series on the lateral internal wall of the spine.

Large round pores (50–90 µm in diameter) almost flush with the coenosteum. They are scattered over the coral surface between the dactylopore spines (Figures 2g, h).

The colonies present sexual dimorphism in both size and position of the ampullae. The female colonies have round ampullae (up to 1 mm in diameter) that protrude from the coenosteum surface (Figure 2l). Small dactylopores are frequently present over the ampullae. The efferent pores are up to 175 µm in diameter and may be visible laterally. Male colonies have smaller, round to elliptical ampullae (up to 500 µm in diameter), which are partially embedded in the coenosteum and almost no detectable at the surface (Figure 2m).

##### Remarks.

In the Antarctic and Sub-Antarctic region 11 *Errina* species have been recorded (Cairns 1983b, 1991). Among them, *Errina fissurata* and *Errina antarctica* are very similar to our specimens in various characters such as coenosteum colour and texture, non-lipped gastropore, and in having two kinds of dactylopores. The characteristic shape of the large dactylopore spines, the shape of the gastrostyles and the dimorphism of the ampullae reported in *Errina fissurata* clearly match with our Antarctic specimens. Moreover, *Errina fissurata* and *Errina antarctica* show a distinct geographical and bathymetric distribution: *Errina fissurata* is described around continental Antarctic, and almost exclusively at > 300 m depth, whereas *Errina antarctica* is only known from South America and usually reported from < 300 m depth.

Our specimens compare favourably with samples described by Cairns (1983a), but differ in having the coenosteal pores distinguishable at the coenosteum surface, the presence of large pores at the surface, and having dactylostyles in the large dactylopores, never described before for this species. To date, the only other *Errina* species described with dactylostyles is *Errina capensis* Hickson, 1912 from South Africa (Cairns and Zibrowius 2013).

#### Errina labrosa

Taxon classificationAnimaliaAnthoathecataStylasteridae

Pica, Cairns & Puce sp. n.

http://zoobank.org/5F5BDBA9-ED95-47AC-8533-71208763C146

Figures 3 , 4 , 5

##### Holotype.

BNHM 1977.8.10.2: four branches of a single colony, *Discovery* Expedition Sta 6, Tristan da Cunha, 3 miles N 30° E of Settlement, 80–140 m depth, 1 February1926 (in ethanol).

##### Paratypes.

MNA 3085: two colonies, Cruise Icefish 2004 (dry); MNA 3087: several fragments (dry).

##### Diagnosis.

The new species has a characteristic abcauline lip, ring palisade and one type of dactylopore with very elongated spines.

##### Description.

**Figure 3.**
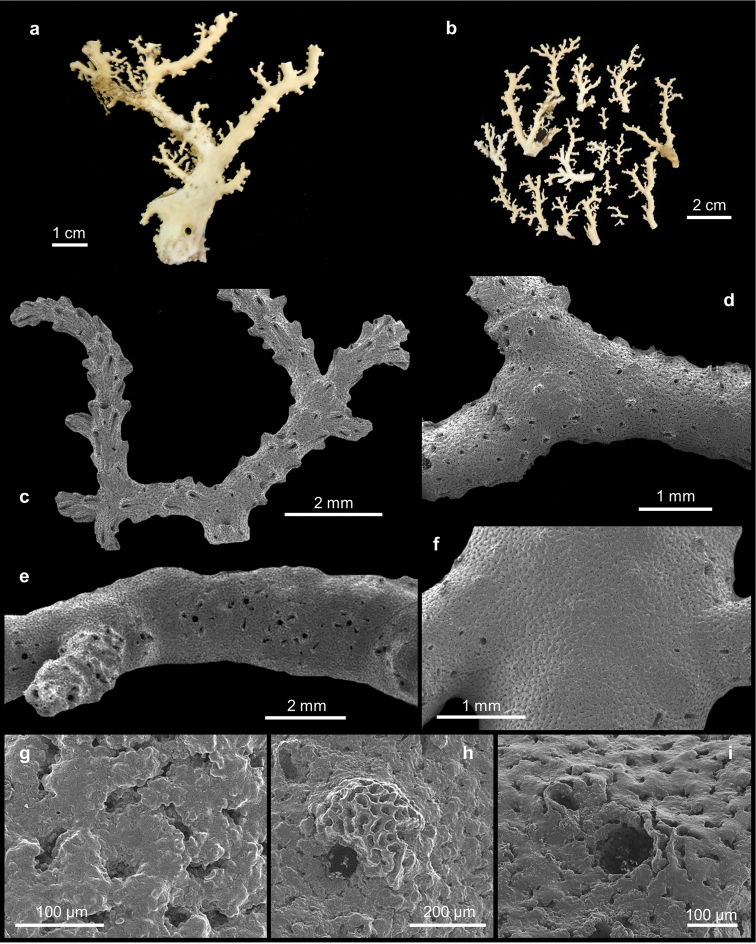
*Errina labrosa* sp. n. **a–b** Holotype. SEM micrographs of **c** spiny branch apex with gastropores and dactylopores uniformly distributed **d** middle portion of the colony **e** lateral view of the colony branch with gastropores are aligned and surrounded by the dactylopores **f** superficial coenosteum without pore **g** texture reticulate-granular **h** gastropore with lip **i** gastropore without lip.

The holotype (Figure 3a) is composed of several branches of a single colony. The larger branch is about 10 cm long and 9 cm wide. The other specimens (Figure 3b) consist of two large colonies and one small and broken colony attached to a black coral. All colonies have an wide base from where flabellate and uniplanar branches arise. Their bases are elliptical in cross-section and the axis of the largest colony is 6 by 4 mm. The apexes are up to 1 mm in diameter. The branches are irregularly sparse and unequal, without anastomosis.

The gastropores and the dactylopores are predominantly concentrated on the terminal branches (Figure 3c) and decrease in abundance towards the base (Figures 3d–f). In this region the pores remain confined to the lateral branch edges where the gastropores are aligned and surrounded by dactylopores characterised by a small spine.

The coenosteum is white-cream in colour and the texture is reticulate-granular with poorly-defined granules (Figure 3g). The strips are irregular in shape, 21–79 µm wide. The surface appears uniformly lumpy.

The gastropores are circular in shape (Figures 3h–i), 80–150 µm in diameter. Predominantly in the apical region of the colony they are bordered by a well-pronounced abcauline lip (Figure 3h). This lip is porous and rounded to rectangular in shape, up to 273 µm wide, and may bear one or two dactylopore spines. The gastropore tubes are cylindrical and shallow, gastrostyles are visible at the surface. The gastrostyle (Figure 4a) has a spindle shape, ornamented with multi-tipped bifurcating spines (Figure 4b) in the apical portion, while the basal region presents a small smooth constriction. They measure 205–289 × 68–114 µm (L:D=2.3–3.4). The wall of the gastropore tube bears a diffuse ring palisade (Figure 4c) composed of irregularly shaped elements up to 20 µm in diameter. Tabulae are absent.

**Figure 4.**
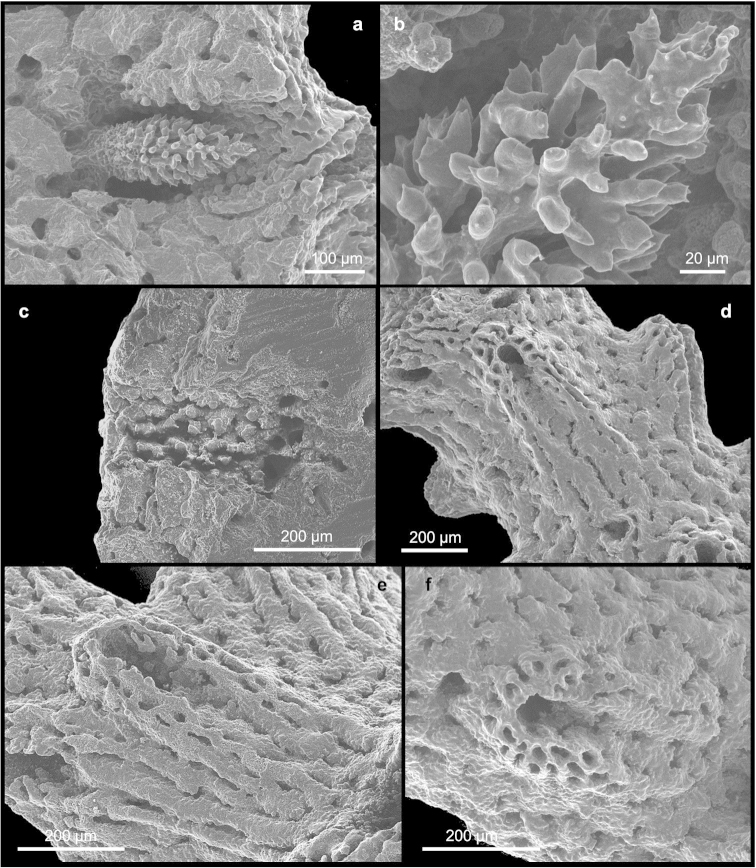
*Errina labrosa* sp. n. SEM micrographs of **a** gastrostyle **b** multitipped bifurcating spines of the gastrostyle **c** diffuse ring palisade **d–f** dactylopores from the apical region to the base.

**Figure 5.**
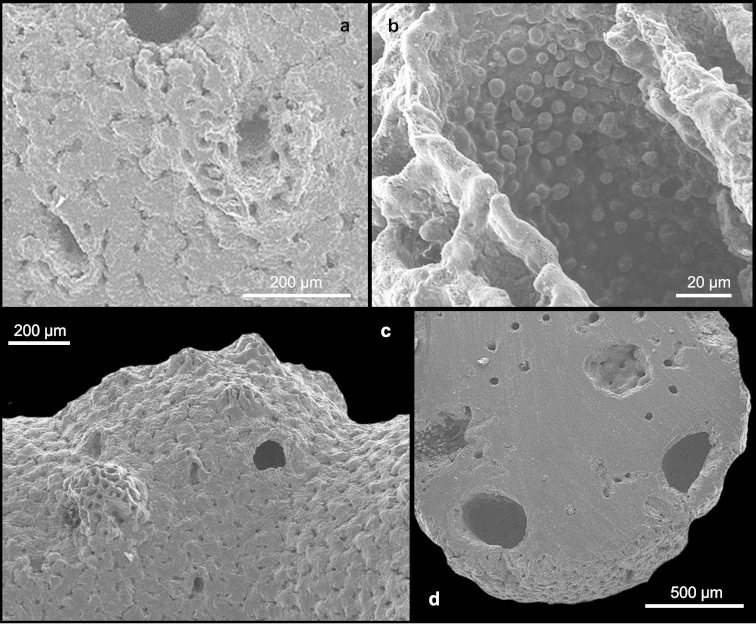
*Errina labrosa* sp. n. SEM micrographs of **a** dactylopore without spine **b** dactylostyle **c–d** ampullae.

Dactylopores are of one kind. In the apical branches they are adcauline and bordered by well-defined spines (Figures 4d–f; 5a). These spines are 104–244 µm long and 158–223 µm wide, truncate, with a long groove (the dactylotome) 300–550 µm long, which is oriented with an angle up to 45° with respect to the branch surface. Laterally, the spines show the same texture as the surface, while the internal wall is characterised by rudimentary dactylostyles, uniformly distributed on all surfaces (Figure 5b). The dactylostyle elements are rounded and 2–6 µm in diameter. The dactylotome is 48–73 µm wide. Proximally, the dactylopore spines are shorter with a smaller groove. In this region the spines are oriented in all directions and they are located mainly on the lateral side of the branches around the gastropores.

The ampullae (Figures 5c–d) are distributed homogenously over the colony. In the apical branches they are external while in the basal branches they are predominantly internal. The female ampullae, 550–842 µm in diameter, are round in shape but appear hemispherical from outside, and may have some dactylopores above them (Figure 5c) and an efferent pore on one side (Figure 5c). The male ampullae, 400–500 µm in diameter, are also round in shape (Figure 5d). No efferent pores were observed for them.

##### Remarks.

Among the 25 known species of *Errina*, the only two other species having a gastropore lip, ring palisade and one type of dactylopore, as in our species, are *Errina cheilopora* Cairns, 1983 and *Errina reticulata* Cairns, 1991. The gastropore lip of these two species projects over the gastropore (Cairns 1991) and therefore differs in shape with respect to the lip observed in our specimens. In addition, both species have large gastropores (180–330 µm and 140–200 µm, respectively) and mainly show a linear-granular texture (Cairns 1991). Our specimens show a characteristic gastropore lip and very elongated dactylopore spines, which distinguish *Errina labrosa* easily from the other species. Moreover, from a geographic point of view, *Errina labrosa* together with *Inferiolabiata spinosa* (see above) represent the first identified stylasterids from the Tristan da Cunha Archipelago in the central part of the South Atlantic Ocean.

*Errina* was reported earlier from Tristan da Cunha by Moseley (1881) based on a specimen dredged by the *Challenger* Expedition (Sta. 135), which was identified as *Errina labiata* (now *Inferiolabiata labiata*). Boschma (1964) doubted this identification because he noted that the openings of the groove spines of the dactylopores are turned toward the basal part of the colony instead of the apical part, as in *Inferiolabiata labiata*. He identified the specimen as *Errina gracilis*. Subsequently Cairns (1983a), reviewing the stylasterids from the Antarctic and Subantarctic region, reidentified the same specimen as Errina (Errina) sp. to because its poor description that did not allow a correct identification and therefore a comparison with our specimen is impossible.

##### Etymology.

From the Latin word *labrum* (meaning lip) for the characteristic abcauline lip of the gastropores.

### Scalpellid barnacles associated with stylasterid corals

**Figure 6.**
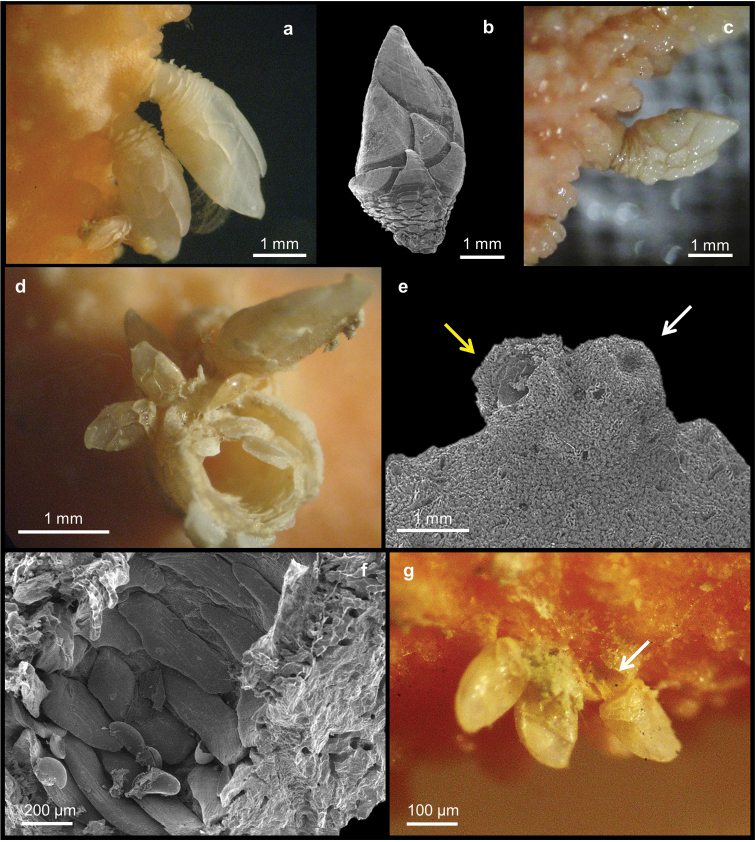
*Errina fissurata*. **a** Specimen with *Trianguloscalpellum* sp. **b** SEM of *Trianguloscalpellum* sp. **c** specimen with Ornatoscalpellum cf. vanhoeffeni **d** peduncular plates in cavity in coral coenosteum **e** SEM of where a barnacle was detached (yellow arrow) next to a superficial bump (white arrow) **f** peduncular scales in a bump **g** young barnacles one with peduncle in a dactylopore (arrow).

The stylasterid *Errina fissurata* is reported as host for two different scalpellids (Table 1). One of them is *Trianguloscalpellum* sp. (Arcoscalpellinae, Figures 6a, b) on specimens MNA 3079 and 3082, while the other is Ornatoscalpellum cf. vanhoeffeni (Scalpellinae, Figure 6c) on specimen BNHM 1977.8.10.26. In all specimens the peduncles are partially covered by a thin layer of coenosteum (Figures 6a, c). By removal of the unburied portion of the peduncle supporting the capitulum, a peduncular cavity within the peduncular plates can be seen penetrating deep into and surrounded by coral coenosteum (Figure 6d). Moreover, on the surface of *Errina fissurata* corals several irregular calcareous bumps can be observed (Figure 6e), which are filled with peduncular scales (Figure 6f). At the base of one of these barnacles, several juvenile barnacles are found (Figure 6d). Similar young specimens have been found at some distance from mature ones attached to the surface or inside dactylopores of the host coral (Figure 6e). The barnacles on *Errina fissurata* are scattered about mainly on the apical region of the corals.

**Figure 7.**
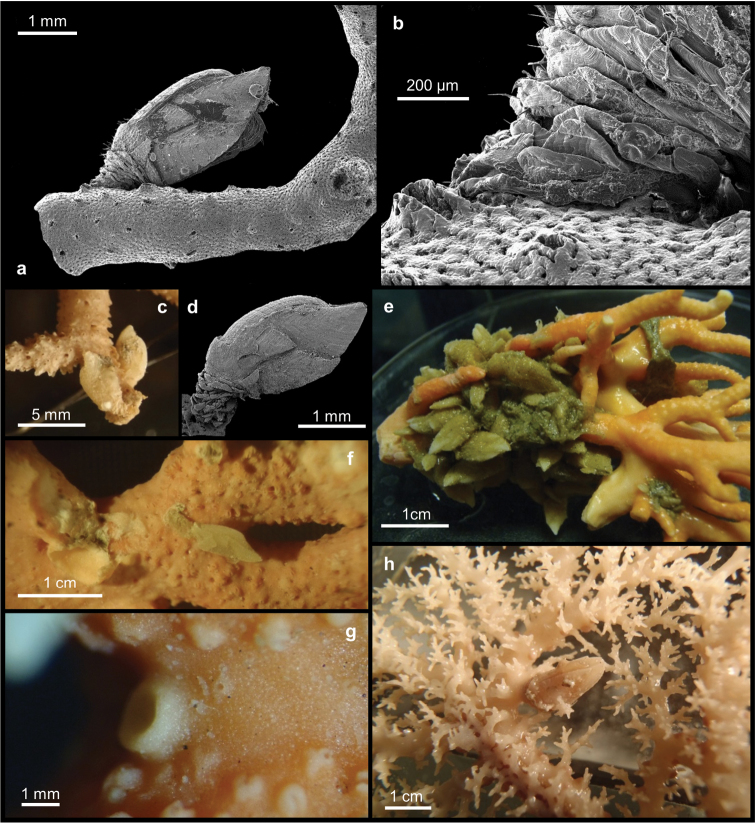
**a–b** Peduncle of *Arcoscalpellum* sp. 1 not covered by coenosteum of *Errina labrosa* sp. n. **c–d** Peduncle of *Arcoscalpellum* sp. 2 not covered by coenosteum in *Inferiolabiata spinosa* and SEM micrograph of same species **e** scalpelline sp. 2 concentrated in the basal portion in *Errina antarctica* **f** peduncle Ornatoscalpellum cf. gibberum attached to but free of coral skeleton in *Errina antarctica* **g** circular bumps with little depressions on the top in *Errina antarctica* **h** scalpelline sp. 3 with peduncle fully and lower margin of capitulum partially covered by the calcareous skeleton of *Stephanohelia* sp.

The new species, *Errina labrosa* is observed in association with *Arcoscalpellum* sp. 1 (Figure 7a, b), while the species *Inferiolabiata spinosa* (Figure 7c) hosted *Arcoscalpellum* sp. 2 (Figure 7d) (Table 1). Specimens of these two cirripedes are attached to the surface of the corals albeit their peduncles are not covered by the coenosteum (Figures 7a–c). Moreover, when such occasional arcoscalpellines are detached nothing more than small depressions are left behind on the coral surface, and no recently settled juveniles were found around them. In *Errina labrosa* individuals of *Arcoscalpellum* sp. 1 are scattered along the length of the colonies, while in *Inferiolabiata spinosa*, *Arcoscalpellum* sp. 2 is mainly distributed over the apical portions (Figure 7c).

Three different scalpellids are found in association with three different specimens of *Errina antarctica*: scalpellid sp.1 on specimens BNHM 1977.8.10.17, scalpellid sp.2 on BNHM 1977.8.10.20 (Figure 7e) and the scalpelline Ornatoscalpellum cf. gibberum on BNHM 1977.8.10.34 (Figure 7f) (Table 1). Individuals of scalpellid sp. 2 (Figure 7e) are concentrated in a mass on the basal portion of the stylasterid. While attached to the coenosteum, their peduncles are not covered by it. On the other hand, individuals of Ornatoscalpellum cf. gibberum are scattered individually over the entire length of the coral colony (Figure 7f). In this species too the peduncle remained uncovered by the coral coenosteum. The same can be said for scalpellid sp. 1, but in this case the coral surface several little bumps are present, circular in shape and with a small depression on the top (Figure 7g). No peduncular scales are visible in the depressions.

The stylasterid *Stephanohelia* sp. has been observed to host a single unidentified scalpellid, sp.3 (Figure 7h) (Table 1) which is located in the middle portion of the coral colony and has its peduncle covered by the coral skeleton up to the capitulum (Figure 7h). No other bumps or scars are observed on the coral surface.

In all cases the barnacles are attached through living tissue of the stylasterids, where their cyprid larvae had settled.

## Discussion

The Stylasteridae of the South Atlantic Ocean, between the Antarctic and the Tropic of Capricorn, with a total of 22 known species, are poorly known (Boschma 1957, Cairns 1983a, Cairns and Zibrowius 2013). They are largely distributed along the coasts of South America and South Africa, except for *Errina labiata* (see remarcks of *Errina labrosa* sp. n.) sampled from Tristan da Cunha during the *Challenger* Expedition (Moseley 1881). This identification was deemed highly questionable by both Boschma (1964) and Cairns (1983a) and currently it is just known as *Errina* sp. (Cairns 1983a). Thus, *Errina labrosa* sp. n. and *Inferiolabiata spinosa* represent the only two identified stylasterid corals known from this locality. This Archipelago is considered part of the Temperate South America Region (Briggs 1974, Briggs and Bowen 2013), where 16 stylasterid species were reported from along the coasts of South America (Cairns 1983a).

Associations between cirripeds and cnidarians are fairly common in both shallow and deep water, involving numerous species of coral-associated barnacles. These include inconspicuous burrowing barnacles or acrothoracicans (Kolbasov 2009), which burrow in limestone and coral skeletons, and the conspicuous shallow-water balanomorph thoracicans of the family Pyrgomatidae (Simon-Blecher et al. 2007, Malay and Michonneau 2014), which settle on and keep pace with the growing coral skeleton (Ross and Newman 1973). Stalked barnacles have been recorded in association with some deep-water cnidarian taxa such as black corals (Molodtsova and Poltarukha 2008, Bo et al. 2012), gorgonians (Buhl-Mortensen and Mortensen 2004a, 2004b, 2005) and scleractinian corals (Newman et al. 2002). There is but one previous record of an association between stylasterid corals and a stalked barnacle (Newman and Ross 1971) even though several taxonomic papers provide information about symbionts of stylasterids (Zibrowius 1981, Goud and Hoeksema 2001, Puce et al. 2009, Pica et al. 2012). General works on stylasterids such, as Cairns (2011) and earlier, only mention barnacles in passing. That leaves Pica et al. (2012) for an overview, upon which the following list is based.

**Subclass Cirripedia Burmeister, 1834** (those previously known associated with shallow as well as deep-water stylastrid corals, plus the scalpellids (Table 1))

**Superorder Acrothoracica Gruvel, 1905**

**Order Lithoglyptida Kolbasov, Newman & Høeg, 2009**

**Family Lithoglyptidae Aurivillius, 1892**

*Armatoglyptes stirni* (Turquier, 1987), off Strait of Gibraltar (Cape Spartel), in *Errina aspera* (L.) and other corals, 90–390 m (Kolbasov 2009).

*Lithoglyptes* s.l. in *Paraerrina decipiens* Brock, 1942 from Mauritius (Zibrowius 1981).

**Order Cryptophialida Kolbasov, Newman & Høeg, 2009**

**Family Cryptophialidae Gerstaecker, 1866**

*Australophialus pecorus* Turquier, 1985, off Strait of Gibraltar (Cape Spartel), in *Errina aspera* (L.) ~200 m and other corals between 20–390 m (Kolbasov 2009).

*Australophialus tomlinsoni* (Newman & Ross, 1971), off Ross Sea and Antarctic Peninsula, in skeletons of *Errina* sp. and other invertebrates, ~400 m (Newman and Ross 1971).

**Superorder Thoracica Darwin, 1854**

**Order Scalpelliformes Buckeridge & Newman, 2006**

**Family Scalpellidae Pilsbry, 1907**

Three species unidentified to genus herein, two on *Errina antarctica*, off Falkland Is. 79–370 m and one on *Stephanohelia* sp. New Caledonia 500 m.

**Subfamily Scalpellinae Pilsbry, 1907**

*Ornatoscalpellum gibberum* (Aurivillius, 1892), off Tierra del Fuego on Errina cf. antarctica (Gray, 1872), 250 m (Newman and Ross 1971).

Ornatoscalpellum cf. gibberum (Aurivillius, 1892) on *Errina antarctica* off Falkland Is, 79–370 m (Aurivillius 1892).

Ornatoscalpellum cf. vanhoeffeni (Gruvel, 1907) on *Errina fissurata* off Daniell Peninsula, Antarctica 438–610 m (Gruvel 1907).

**Subfamily Arcoscalpellinae Zevina, 1978**

*Trianguloscalpellum* sp., on *Errina fissurata* (Gray, 1872) from off Daniell Peninsula, Antarctica 438–610 m (Gray 1872).

*Arcoscalpellum* sp. 1 and 2, one on *Errina labrosa* sp. n., the other on *Inferiolabiata spinosa* Cairns, 1991, all from Tristan da Chuna, 80–140 m (Cairns 1991).

**Order Sessilia Lamarck, 1818**

**Suborder Verrucomorpha Pilsbry, 1916**

**Family Verrucidae Darwin, 1854**

*Verruca* s.l. on *Errina dabneyi* (De Pourtalès, 1871) from Açoc Seamount at 400 m (Braga-Henriques et al 2010, Braga-Henriques pers. comm.).

**Suborder Balanomorpha Pilsbry, 1916**

**Family Pachylasmatidae Utinomi, 1968**

*Pachylasma giganteum* (Philippi, 1836), Strait of Messina to off W. Africa, facultative with *Errina aspera* (L.), at shelf break, 150–200 m (Darwin 1854, Zibrowius 1981, Fredj and Giermann 1982, Di Geronimo and Fredj 1987, Zibrowius and Cairns 1992, Foster and Buckeridge 1995, Salvati et al. 2010).

**Family Archaeobalanidae Newman & Ross, 1976**

*Solidobalanus enbergi* (Pilsbry, 1921) shallow water, facultative on a stylasterid; possibly a senior synonym or sibling of *Armatobalanus nefrens* (see Young and Shimek 1982).

*Armatobalanus nefrens* (Zullo, 1963) shallow water, generally in *Stylaster californicus* (Verrill, 1866) and *Errinopora pourtalesi* (Dall, 1884).

**Family Pyrgomatidae Gray, 1825**

?*Pyrgoma* sp. on *Stylaster ramosus* Broch, 1947 from Tanzania, shallow water, possibly a pyrgomatid, but not likely a *Pyrgoma* species as presently known.

?*Pyrgoma* sp. on *Stylaster scabiosus* Broch, 1935 from Mauritius, shallow water, possibly a pyrgomatid, but not likely a *Pyrgoma* species as presently known.

**Family Balanidae Leach, 1806**

*Balanus nubilus* Darwin, 1854, intertidal and shallow water, occasionally all but overgrown by a *Stylaster* sp. in British Columbia.

Only two previously recorded cases of associations between scalpellomorphs and cnidarians provided with a hard calcareous skeleton are known, one illustration of *Ornatoscalpellum gibberum* (Aurivillius, 1892) on *Errina* sp., likely *Errina antarctica* (Newman and Ross 1971, Plate XIII), and the other of calanticids of the *Scillaelepas* complex on scleractinians from North Atlantic and New Zealand (Newman et al. 2002).

The scapellids reported here have been found in different positions on the stylasterid colonies and also in different stages of development. On the colony of *Errina fissurata* and *Errina antarctica* a morphological reaction to the presence of the symbiont has been observed. In fact, the cirriped induces or allows the production of a calcareous collaret surrounding the lower portion of its peduncle. A similar arrangement was reported by Newman et al. (2002) in the association between calanticid scalpellomorph and the scleractinian, *Lophelia pertusa*, where it settled in the coral calices. The presence of the barnacle caused coral skeleton or coenosteum to grow up around the base of the barnacle peduncle in much the same manner as seen here in some of the stylasterids. Regarding black corals, Bo et al. (2012) confirmed previous reports that the barnacle’s settling induces a skeletal reaction resulting in an outgrowth of skeletal tissue with modified spines, and this can apparently also occur in cases involving some stylasterids-inhabiting barnacles (Figure 7h).

It was observed that when a cirriped accidentally detaches or dies, the hydroid partially plugs the gall with skeleton material which remains clearly visible as a bump-like scar. On the internal face of the bump the peduncular scales of the barnacle are still visible (Figures 6e, f). Similar attachment scars consisting of scleractinian skeleton material are reported by Newman et al. (2002, Figure 3 B and C) but here the cavity was only partially coated, not filled by the coral.

Several scalpellid individuals in different early stages of growth were found on colonies of *Errina fissurata*. The cyprid larvae settled on the peduncle of established individuals as well as directly on the coral surface. In the latter case the cyprid larvae may settle inside dactylopore openings.

## Conclusions

The study of the Stylasteridae corals from European museum collections did not only reveal various associations between stylasterid corals and pedunculate barnacles, but also allowed the description of *Errina labrosa* sp. n., which also participated in this association.

Overall, eight scalpellid species are recorded in association with five stylasterid coral species belonging to at least three genera. Our study suggests a lack of host specificity in this association. In fact, several barnacle species are found to be associated with *Errina fissurata* and *Errina antarctica*, but it appears that in a single host coral colony only a single barnacle species can be represented. Although no specific association has been found, different grades of reaction to the symbiosis have been recorded in the coral. In *Errina labrosa* and *Inferiolabiata spinosa* no reaction has been observed, while in *Errina fissurata* and *Stephanohelia* sp. the coenosteum covered the peduncles of both observed barnacles species. *Errina antarctica* shows both kinds of interaction. This suggests that the reaction of stylasterid corals depends on the barnacle species, but because a wide range of sizes (ages) of each species was not available, further investigations are needed to test this hypothesis. The symbiosis between stylasterid corals and scalpellid barnacles, albeit relatively rare, is largely confined to vulnerable marine ecosystems of the Southern Ocean (Jones and Lockhart 2011). Such biodiversity is not spread evenly across the ocean floor but follows complex patterns determined by climate, geology and evolutionary history.

## Supplementary Material

XML Treatment for Stephanohelia

XML Treatment for Stephanohelia sp.

XML Treatment for Inferiolabiata

XML Treatment for Inferiolabiata spinosa

XML Treatment for Errina

XML Treatment for Errina antarctica

XML Treatment for Errina fissurata

XML Treatment for Errina labrosa
